# Elevated serum TREM-1 is associated with periodontitis and disease activity in rheumatoid arthritis

**DOI:** 10.1038/s41598-021-82335-9

**Published:** 2021-02-03

**Authors:** Nevsun Inanc, Gonca Mumcu, Meryem Can, Meral Yay, Angelika Silbereisen, Daniel Manoil, Haner Direskeneli, Nagihan Bostanci

**Affiliations:** 1 Division of Rheumatology, Department of Internal Medicine, School of Medicine, Marmara University, Istanbul, Turkey; 2 Department of Health Management, Faculty of Health Sciences, Marmara University, Istanbul, Turkey; 3 Division of Rheumatology, Department of Internal Medicine, School of Medicine, Medipol University, Istanbul, Turkey; 4 Department of Statistics, Mimar Sinan Fine Arts University, Istanbul, Turkey; 5 Section of Periodontology and Dental Prevention, Division of Oral Diseases, Department of Dental Medicine, Karolinska Institutet, Alfred Nobels alle 8, 14104 Huddinge, Stockholm Sweden

**Keywords:** Biomarkers, Pathogenesis, Rheumatology, Risk factors

## Abstract

The triggering receptor expressed on myeloid cells 1 (TREM-1) and peptidoglycan recognition protein 1 (PGLYRP1) are involved in the propagation of inflammatory responses. This study investigated whether serum levels of TREM-1 and PGLYRP1 correlate with periodontitis in rheumatoid arthritis (RA) patients. A total of 154 non-smoking participants with RA (n = 55, F/M: 41/14), Behçet´s disease (BD, n = 41, F/M: 30/11) and healthy controls (HC, n = 58, F/M: 40/18) were recruited. Serum and saliva were collected, the 28-joint disease activity score (DAS-28) was calculated and dental/periodontal measurements were recorded. Serum TREM-1 and PGLYRP1 levels were measured by ELISA and salivary bacterial DNA counts by quantitative polymerase chain reaction. TREM-1 and PGLYRP1 levels were higher in RA (166.3 ± 94.3; 155.5 ± 226.9 pg/ml) than BD (102.3 ± 42.8; 52.5 ± 26.3 pg/ml) and HCs (89.8 ± 55.7; 67.4 ± 37.3 pg/ml) (p < 0.05). In RA, periodontitis was associated with increased TREM-1 and PGLYRP1 levels (p < 0.05), yet in patients under methotrexate TREM-1 levels were lower. TREM-1 correlated with C-reactive protein (CRP) levels, DAS-28 and erythrocyte sedimentation rate, whereas PGLYRP1 positively correlated with CRP. RA patients displayed 3.5-fold higher salivary bacterial DNA counts than HCs. Increased serum TREM-1 levels correlated with PGLYRP1, CRP and DAS-28-ESR in RA patients with periodontitis.

## Introduction

The triggering receptor expressed on myeloid cells 1 (TREM-1) is a transmembrane receptor of the immunoglobulin superfamily mainly expressed on neutrophils, monocytes and macrophages^1–4^. TREM-1 works in synergy with Toll-like receptor signaling to trigger and amplify inflammatory immune responses^2,5,6^. In addition to membrane-bound TREM-1, TREM-1 can be shed into body fluids as soluble TREM-1 through matrix metalloproteinase-mediated proteolytic cleavage^2,7^. TREM-1 has been investigated as a biomarker and therapeutic target in both infectious diseases, such as pneumonia or sepsis^8^, and non-infectious inflammatory diseases, such as atherosclerosis^6^, Behçet´s disease (BD)^9^ or rheumatoid arthritis (RA)^6,10^.

RA is a systemic autoimmune disease characterized by chronic inflammation of the synovium, bone and cartilage, involving a complex interplay between genetic and environmental factors that are responsible for the disease development and perpetuation of chronic inflammation^11^. Previous studies have shown that TREM-1 levels in serum^12^, synovial fluid^13^ and plasma^14,15^ are elevated in RA compared to non-RA patients. Furthermore, treatment-naïve RA patients were shown to bare higher serum levels of TREM-1 compared to RA patients treated by disease-modifying anti-rheumatic drugs (DMARDs)^12^. Positive correlations were also identified between serum TREM-1 levels and RA disease activity (28-joint disease activity score—DAS-28), C‐reactive protein (CRP) and levels of anti-citrullinated protein antibodies (ACPA), a hallmark of RA pathogenesis^12,16^. Similarly, plasma TREM-1 levels were also positively correlated with DAS-28, CRP, erythrocyte sedimentation rate (ESR), white blood cell counts, neutrophil counts, and tumor necrosis factor-α (TNF-α) levels^15^.

Peptidoglycan recognition protein 1 (PGLYRP1) is an antimicrobial protein present in polymorphonuclear leukocyte granules and secreted upon degranulation to counteract microbial infections. As ligand of TREM-1, PGLYRP1 along with a microbial stimulus, can activate the TREM-1 signaling pathway resulting in a pro-inflammatory immune response^5,6^. Previous investigations revealed an association between single nucleotide polymorphisms of the PGLYRP1-encoding gene and RA^17^. Furthermore, serum levels of PGLYRP1 were shown to be associated with rheumatoid factor (RF) and ACPA in RA and therefore suggested as a potential biomarker for RA^18^. However, so far, the role of PGLYRP1 in RA is still poorly understood.

Periodontitis is a biofilm-driven chronic inflammatory disease during which a dysbiotic periodontal microbiota induces adverse immune responses that further lead to the destruction of tooth-supporting tissues and ultimately tooth loss^19–21^. Several studies have investigated TREM-1 and PGLYRP1 in the context of periodontal disease and recently TREM-1 has been suggested as a candidate marker for new treatment strategies against periodontal diseases^22,23^. Elevated TREM-1 levels were observed in saliva, serum and gingival crevicular fluids (GCF) in patients with periodontitis, and TREM-1 serum and saliva levels correlated positively^24–27^. PGLYRP1 levels were elevated in saliva and GCF of patients with periodontitis, and initial periodontal therapy decreased PGLYRP1 levels in these patients with a stronger effect in non-smokers^27–29^. Furthermore, TREM-1 and PGLYRP1 have been investigated in the context of the periodontal and systemic disease link^28,30–32^. In this regard, higher levels of salivary TREM-1 and PGLYRP1 were associated with poorer oral health in chronic kidney disease patients with concomitant periodontitis, and a positive correlation was found between salivary TREM-1 and PGLYRP1 levels^28^.

Periodontitis and RA are both chronic destructive inflammatory diseases that share common features in their pathogenesis, including the inflammatory destruction of bone tissue. Additionally, there is evidence demonstrating an epidemiological link between RA and periodontitis^33^. Yet, the mechanisms underlying this association remain to be elucidated. In this consideration, a potential contribution of TREM-1 and PGLYRP1 as underpinning effectors has yet not been addressed to our knowledge. Therefore, further studies and detailed statistical analysis are required to dissect the mechanisms underlying the association between RA and periodontitis in the achievement of therapeutic goals in RA. A mediation analysis (MA) could be a suitable option to evaluate this association. MAs commonly serve to statistically explore the mechanisms that underlie the relationship between an independent and a dependent variable, considering a potential indirect effect mediated by a so-called mediator variable^34,35^.

The present study aimed to investigate potential associations that exist in RA patients between serum levels of TREM-1, its ligand PGLYRP1, periodontitis and indicators of RA disease activity. We additionally compared these parameters relative to the uptake of methotrexate, a drug that has displayed significant efficacy in the treatment of RA and that was also shown to positively impact the outcome of periodontal therapy^36,37^.

## Methods

### Study cohort

In this cross-sectional study, 55 patients with RA (F/M: 41/14, mean age: 48.8 ± 11.4 years), 41 patients with Behçet disease (BD, F/M: 30/11, 38 ± 9.4 years) and 58 healthy control participants (HC, F/M: 40/18, 41 ± 12 years) were recruited from the Division of Rheumatology, School of Medicine, Marmara University, Turkey. Current investigations conformed to ethical principles of the Declaration of Helsinki and were approved by the Ethical Committee of Marmara University Medical School (MAR-YÇ-2009-0295). All participants enrolled gave their informed consent.

RA was diagnosed according to 1987 American College of Rheumatology criteria^38^. RA disease activity was assessed using the 28-joint disease activity score based on erythrocyte sedimentation rate (DAS-28-ESR). RA patients were treated by conventional synthetic disease modifying antirheumatic drugs (csDMARDs) for more than 6 months, none was administered biological therapy. For this study, these patients were categorized based on whether methotrexate (MTX) was included in their therapeutic regimen or not: MTX (n = 25) and non-MTX (n = 30) groups.

Patients affected by BD as a chronic and relapsing systemic inflammatory disorder were additionally recruited herein to serve as a disease control group associated with oral health impairment^34^. BD patients were diagnosed according to the International Study Group for Behçet’s disease criteria^39^ and the cohort was characterized by a disease severity score of 3.9 ± 1.4 (mucocutaneous involvement: n = 31; i.e. 75,6% vs. major organ involvement: n = 10; i.e. 24,4%). The healthy control (HC) group was recruited from family-unrelated individuals who were accompanying patients attending the Rheumatology outpatient clinic. These individuals were matched for socio-economic status. Exclusion criteria included the presence of cardiovascular and respiratory diseases, diabetes mellitus and current pregnancy or lactation. Participants who received antibiotics, or any other medication that may potentially affect their periodontal status, within 6 months prior to their enrollment were also excluded. In this regard, patients receiving prednisone > 10 mg/day, were excluded from the study as the treatment may have effects on their oral microbiota. Finally, current smokers were also excluded.

### Oral health parameters, whole saliva collection and salivary bacterial load

Oral health was evaluated using various dental and periodontal indices. The number of decayed, missing and filled teeth were used to calculate the so-called DMFT index, which provides an estimation of how much the dentition has become affected by dental caries until the day of examination^40^. Periodontal parameters including plaque index (PI); a measure of the amount of dental plaque on teeth surfaces^41^, bleeding on probing (BOP); a measure of gingival inflammation, probing pocket depth (PPD) and clinical attachment loss (CAL), were recorded at six sites around each tooth. All teeth except third molars were included in the oral examination^42^. Participants displaying ≥ 2 interproximal sites with CAL values ≥ 4 mm (not on the same tooth) were diagnosed as periodontitis positive^42^.

Additionally, all participants provided unstimulated saliva. Saliva samples were collected between 9 and 11 a.m. Participants were instructed not to eat, drink, brush, floss or apply any other measures of oral hygiene within 2 h preceding saliva collection. Participants rinsed their oral cavity with distilled water during two minutes just before sampling, and saliva was then collected by 15 min-passive drooling into graduated test tubes. Total bacterial counts in saliva were quantified by means of quantitative polymerase chain reaction (qPCR) using validated protocols and primers previously described^43,44^. Because non-shedding tooth surfaces are an important source of bacteria detected in saliva^45^, total bacterial DNA counts were normalized against the number of natural teeth present in the oral cavity.

### Serum levels of TREM-1 and PGLYRP1

Collected fasting peripheral blood was used to measure serum levels of TREM-1 and PGLYRP1. Measurements were performed using dedicated ELISA kits (DuoSet, R&D Systems, Abingdon, UK) according to the instructions of the manufacturer as described earlier^46^. The Westergren method was used to measure the ESR (mm/h) and nephelometry was employed for CRP measurements (mg/l).

### Statistical analysis

Analyses were carried out using SPSS 26.0 statistic program (SPSS Inc, Chicago, IL, USA). As the data set demonstrated a non-normal distribution (Kolmogorov–Smirnov test, p < 0.05), Kruskal–Wallis tests were employed for statistical analyses involving the three groups, whereas Mann–Whitney tests were employed to compare pair of groups or variables. Spearman’s rank coefficients were computed to identify correlations. Statistical significance was set at a 95% confidence interval (p < 0.05).

In this study, a mediation analysis (MA) was carried out on RA patients to evaluate both direct and indirect causal effects of selected independent variables on a dependent variable. PROCESS macro was adopted in SPSS 26.0 for the MA. Specifically, the effect of periodontitis, as an independent variable, was assessed on TREM-1 serum levels as the dependent variable. PGLYRP1 serum levels were included as the possible mediator variable in this analysis. In the MA, a bootstrap resampling up to 5000 replications was applied to generate a 95% CI and thereby allow accurate estimation of mediation effects. The mediation effect was considered to be present since the lower and upper intervals did not contain zero^34,35^.

## Results

### Clinical and demographic parameters

Table 1 presents an overview of the demographic and clinical parameters of the participants recruited herein. No gender differences were observed between groups (p > 0.05). Disease duration was slightly lower in RA (7.9 ± 5.9 years) than in BD (10.5 ± 6.9 years) (p = 0.038) whereas the mean age was higher in RA patients (48.8 ± 11.4) as compared to controls (BD: 38 ± 9.4 years and HC: 41 ± 12 years) (p = 0.000). RA patients displayed a mean DAS-28-ESR score of 4.3 ± 1.5 and 45.5% of RA patients were under MTX treatment (Table 1). In addition, these patients exhibited mean CRP and ESR measurements of 9.4 ± 9.3 (mg/l) and 28.2 ± 18.1 (mm/h), respectively.

**Table 1:** Overview of the clinical characteristics of the study cohort. On the right side of the table, the two last columns display p values from pair of groups comparisons (Mann–Whitney, p = 0.05).

	RA (n = 55)		BD (n = 41)		HC (n = 58)		RA-BD	RA-HC
	n	%	n	%	n	%		
Female	41	74.5	30	73.2	40	69	0.88	0.512
Male	14	25.5	11	26.8	18	31		
Total	65	100	43	100	59	100		
Non-MTX treatment	30	54.5						
MTX treatment	25	45.5						
Total	55	100						

	Mean	SD	Mean	SD	Mean	SD	RA-BD	RA-HC
Age (years)	48.84	11.42	37.98	9.35	40.8	11.98	**0.000**	**0.000**
Disease duration (years)	7.87	5.88	10.53	6.88	–	–	**0.038**	**–**
DAS-28-ESR	4.3	1.52	–	–	–	–	–	–
CRP (mg/L)	9.39	9.33	–	–	–	–	–	–
ESR (mm/h)	28.2	18.08	–	–	–	–	–	–

Significant p values are bolded.

*RA* rheumatoid arthritis, *BD* Behçet’s disease, *HC* healthy control, *DAS-28-ESR* 28-joints disease activity score based on erythrocyte sedimentation rate, *CRP* C-reactive protein, *ESR* erythrocyte sedimentation rate, *MTX* methotrexate.

### Oral health parameters

**Figure 1:**
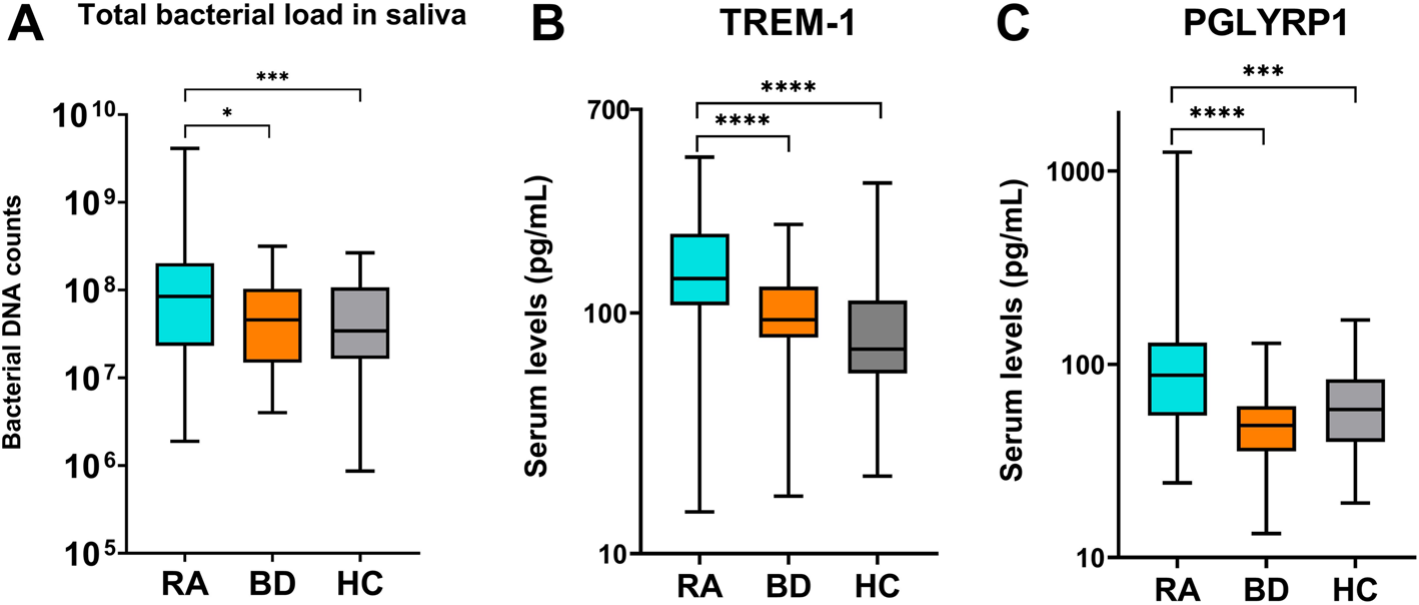
Comparisons among the three groups. (**A**) total bacterial DNA counts in saliva are compared between pair of groups (Mann–Whitney, p = 0.05). (**B**) serum TREM-1 levels are compared between pair of groups (Mann–Whitney, p = 0.05). (**C**) serum PGLYRP1 levels are compared between pair of groups (Mann–Whitney, p = 0.05). Boxplots show the first and third quartile (top and bottom edges of the rectangle) divided by the median. Whiskers correspond to the highest and lowest values. A base-10 log scale is applied on the y-axis. ***p ≤ 0.001, ****p ≤ 0.0001. *RA* rheumatoid arthritis, *BD* Behçet’s disease, *HC* healthy control.

Prevalence of periodontitis was higher in the RA group (38%) as compared to BD (22%) and HC (7%) groups (p < 0.05) (Table 2). RA patients demonstrated significantly higher DMFT indices as compared to BD and HC participants (RA; 9.3 ± 7.1, BD; 5.7 ± 4.1 and HC; 3.9 ± 4) (p < 0.05) (Table 2). When considering the number of missing teeth, RA patients presented with less teeth than the BD and HC groups (RA; 19.3 ± 7.2, BD; 24.1 ± 3.7 and HC; 25.5 ± 3.7) (p < 0.000) (Table 2). On average, saliva from RA patients displayed approximately 3.5-fold higher total bacterial DNA counts than BD and HC participants (Fig. 1A) (p < 0.05).

**Table 2:** Overview of oral health parameters recorded in the study cohort. On the right side of the table, the two last columns display p values from pair of groups comparisons (Mann–Whitney, p = 0.05).

	RA (n = 55)		BD (n = 41)		HC (n = 58)		RA-BD	RA-HC
	Mean	SD	Mean	SD	Mean	SD		
Number of teeth	19.31	7.24	24.12	3.74	25.47	3.73	**0.000**	**0.000**
DMFT index	9.32	7.1	5.71	4.1	3.86	3.96	**0.011**	**0.000**
PI (Q-H)	1.25	1.13	1.3	1.1	0.85	0.83	0.716	0.055
BoP %	45.54	40.58	46.01	38.42	30.34	38.48	0.970	0.062
PPD (mm)	2.83	0.57	2.77	0.4	2.5	0.35	0.755	**0.004**
CAL (mm)	3.49	1.12	3.13	1.3	2.7	0.59	0.301	**0.000**

	n	%	n	%	n	%		
Periodontitis (−)	34	61.82	32	78.05	54	93.1	0.091	**0.000**
Periodontitis (+)	21	38.18	9	21.95	4	6.9		

Significant p values are bolded.

*RA* rheumatoid arthritis, *BD* Behçet’s disease, *HC* healthy control, *DMFT* decayed, missing and filled teeth index, *PI (Q–H)* plaque index (Quigley–Hein), *BoP* bleeding on probing, *PPD* probed pocket depth, *CAL* clinical attachment loss.

### Serum levels of TREM-1 and its ligand PGLYRP1

Serum TREM-1 and PGLYRP1 levels were significantly higher in the RA group (166.3 ± 94.3 pg/ml; 155.5 ± 226.9 pg/ml, respectively) as compared to BD (102.3 ± 42.8 pg/ml; 52.5 ± 26.3 pg/ml) and HC groups (89.8 ± 55.7 pg/ml; 67.4 ± 37.3 pg/ml) (p < 0.05) (Fig. 1B,C). In addition, positive correlations between serum levels of TREM-1 and PGLYRP1 were identified in all groups (r: 0.5, p = 0.000 in RA; r: 0.6, p = 0.000 in BD and r: 0.5, p = 0.000 in HC) (Table 3).

**Table 3:** Spearman’s correlation analyses between serum levels of TREM-1, PGLYRP1 and disease-associated factors.

	RA		BD		HC	
	r	p	r	p	r	p
TREM-1 and PGLYRP1	**0.511**	**0.000**	**0.58**	**0.000**	**0.529**	**0.000**
TREM-1 and DAS-28-ESR	**0.284**	**0.036**	**–**	**–**	**–**	**–**
TREM-1 and ESR	**0.324**	**0.016**	**–**	**–**	**–**	**–**
TREM-1 and CRP	**0.388**	**0.003**	**–**	**–**	**–**	**–**
TREM-1 and DMFT	0.194	0.155	**0.394**	**0.011**	0.071	0.598
PGLYRP1 and DAS-28-ESR	0.164	0.23	**–**	**–**	**–**	**–**
PGLYRP1 and ESR	0.241	0.076	**–**	**–**	**–**	**–**
PGLYRP1 and CRP	**0.334**	**0.013**	**–**	**–**	**–**	**–**
PGLYRP1 and DMFT	0.225	0.099	**0.362**	**0.02**	− 0.063	0.643
CRP and DMFT	0.241	0.076	**–**	**–**	**–**	**–**

Significant correlations are bolded.

*RA* rheumatoid arthritis, *BD* Behçet’s disease, *HC* healthy control, *DAS-28-ESR* 28-joints disease activity score based on erythrocyte sedimentation rate, *DMFT* decayed, missing and filled teeth index, *ESR* erythrocyte sedimentation rate, *CRP* C-reactive protein.

### Correlations between TREM-1, PGLYRP1 and clinical disease activity in patients with RA

We further analyzed how serum levels of TREM-1 and PGLYRP1 correlated with other clinical and serological indicators of RA activity (Table 3). TREM-1 appeared to positively correlate with DAS-28-ESR (r: 0.3, p = 0.036), ESR (r: 0.3, p = 0.01), and CRP levels (r: 0.4, p = 0.003), whereas PGLYRP1 demonstrated positive correlation with CRP (r: 0.3, p = 0.013) (Table 3).

### Serum TREM-1 and PGYLRP1 levels are lower in RA patients on methotrexate

**Figure 2:**
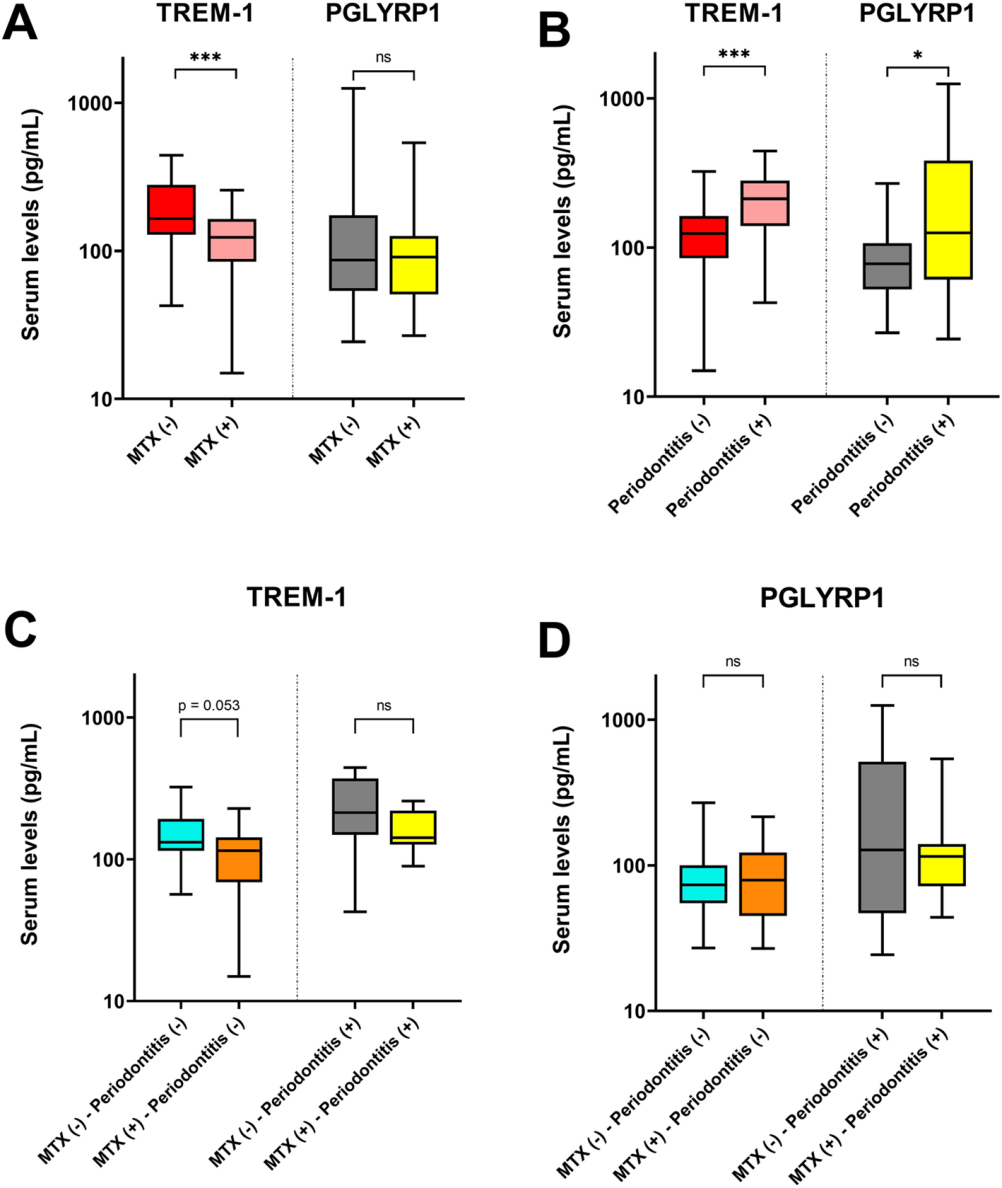
TREM-1 and PGLYRP1 serum levels in RA patients. (**A**) serum levels of TREM-1 and PGLYRP1 are compared between RA patients with and without MTX in their therapeutic regimen. (**B**) serum levels of TREM-1 and PGLYRP1 are compared between RA patients affected or not by periodontitis. (**C**) and (**D**) serum levels of TREM-1 and PGLYRP1 are compared between subgroups of RA patients clustered based on MTX use and presence of periodontitis. Boxplots show the first and third quartile (top and bottom edges of the rectangle) divided by the median. Whiskers correspond to the highest and lowest values. A base-10 log scale is applied on the y-axis. *p ≤ 0.05, **p ≤ 0.01, ***p ≤ 0.001. *MTX* methotrexate, *ns* non-significant.

In the RA cohort, 25 out of 55 patients were under methotrexate treatment (MTX; 45.5%) whereas the rest of the patients were under other csDMARDs (non-MTX; 54.5%). MTX-treated patients displayed significantly lower serum levels of TREM-1 than non-MTX patients (127.7 ± 60.5 pg/ml vs. 198.5 ± 105.7 pg/ml) (p = 0.007) (Fig. 2A). In contrast, no differences in PGLYRP1 levels were observed between MTX- and non-MTX-treated patients (Fig. 2A).

### Serum TREM-1 and PGYLRP1 levels are higher in RA patients with periodontitis

RA patients diagnosed with periodontitis displayed significantly higher TREM-1 and PGLYRP1 levels as compared to patients without periodontitis (TREM-1; p = 0.001 and PGLYRP1; p = 0.037) (Fig. 2B). On an indicative basis, these associations between periodontitis, TREM-1 and PGLYRP1 levels were not significant in HC and BD groups (p > 0.05), although a similar trend could be observed in HC participants (Supplementary Fig. 1A,B). RA patients affected by periodontitis also displayed higher serum levels of TREM-1 (219.2 ± 108 pg/ml) and PGLYRP1 (265.7 ± 336.8 pg/ml) as compared to periodontitis-affected BD patients (TREM-1; 107.9 ± 47.7 pg/ml and PGLYRP1; 53.8 ± 22.6 pg/ml) (p = 0.001 and p = 0.009 for TREM-1 and PGLYRP1 respectively).

### Serum TREM-1 and PGLYRP1 levels in RA patients according to MTX use and periodontitis

We further analyzed serum levels of TREM-1 and PGLYRP1 in relation to both MTX use and diagnosis of periodontitis. For these analyses, RA patients were first clustered according to the presence or absence of periodontitis, and then according to the use of MTX to eventually form 4 subgroups: (1) No-MTX use—no periodontitis, (2) MTX use—no periodontitis (3) No-MTX use—periodontitis and (4) MTX use—periodontitis (Fig. 2C,D).

The highest serum levels of TREM-1 (244.2 ± 119.2 pg/ml) and PGLYRP1 (317.8 ± 390.6 pg/ml) were observed in non-MTX patients affected by periodontitis. In contrast, the lowest levels of TREM-1 (111.6 ± 53.6 pg/ml) and PGLYRP1 (86.6 ± 52.3 pg/ml) were observed in MTX-treated patients devoid of periodontitis (Fig. 2C,D).

In RA patients without periodontitis, TREM-1 levels tended to be lower in relation to the use MTX-treatment, yet this observation only bordered statistical significance (MTX TREM-1: 111.6 ± 53.6 pg/ml vs. non-MTX TREM-1: 158.5 ± 75.2 pg/ml) (p = 0.053) (Fig. 2C). When periodontitis was present, no significant variation in TREM-1 serum levels was observed in relation to MTX-treatment (MTX TREM-1: 169.1 ± 60.7 pg/ml vs. non-MTX TREM-1: 244.2 ± 119.2 pg/ml) (p = 0.117) (Fig. 2C). In the case of PGLYRP1, no significant variations in serum levels were observed neither in relation to periodontitis nor MTX use (Fig. 2D).

### Mediation analyses for serum TREM-1 levels

**Figure 3:**
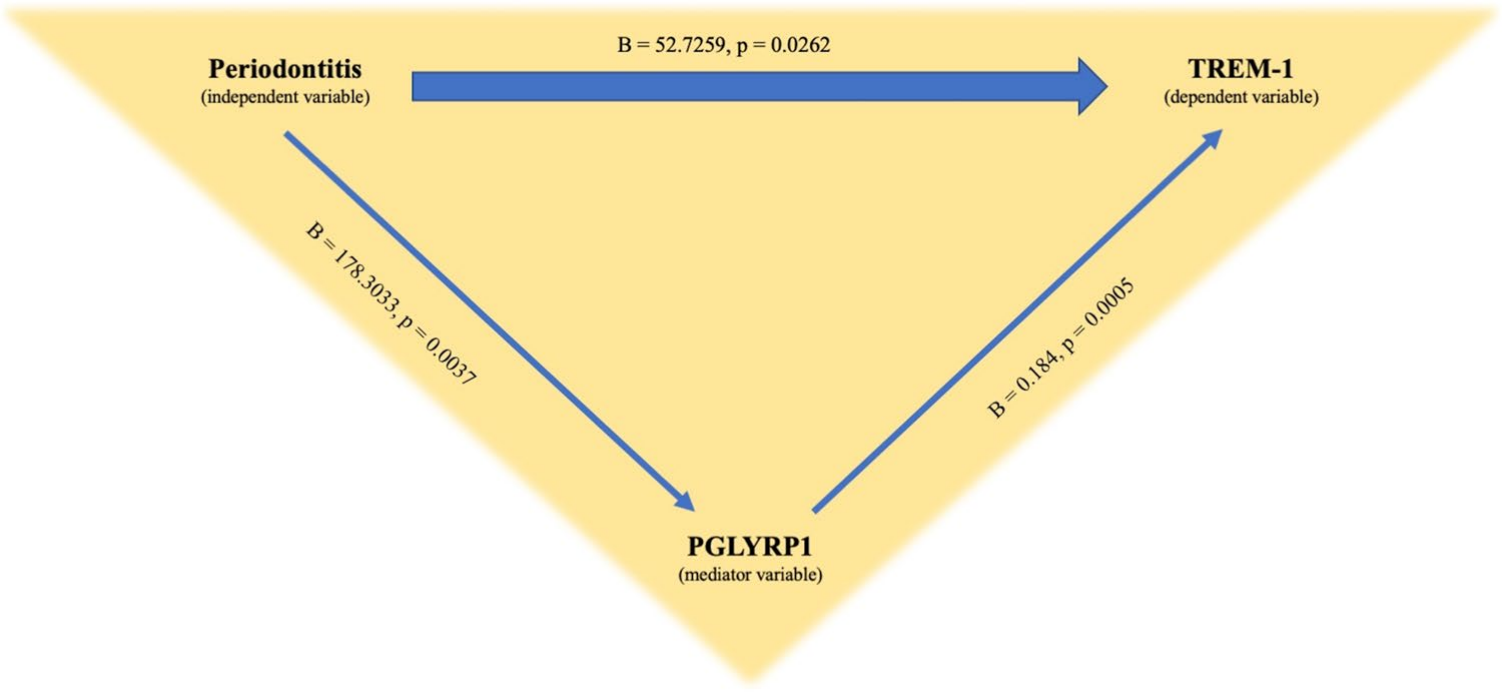
Scheme of the mediation analysis that illustrates the direct effect of “periodontitis” as independent variable on the dependent variable “TREM-1”, as well as the indirect effect of “PGLYRP1” as the mediator variable.

The mediation analyses showed that serum TREM-1 levels were directly mediated by the presence of periodontitis (B = 52.7259, p = 0.0262) and indirectly mediated via PGLYRP1 serum levels in patients with RA (B = 0.1840, p = 0.0005) (Fig. 3). Moreover, periodontitis was shown to affect the levels of PGLYRP1 (B = 178.3033, p = 0.0037) (Fig. 3). Both direct and indirect effects on serum TREM-1 levels were present based on 5000 bootstrap samples, with percentile confidence intervals of 6.49–98.97 and 4.71–69.08, respectively.

## Discussion

It is known that the pathogenesis of RA and periodontitis share common features and increasing evidence supports an epidemiological link between these two diseases^33,47^, although the underpinning mechanisms remain unclear. To the best of our knowledge, this is the first time that TREM-1 and PGLYRP1 were investigated in RA patients with concomitant periodontitis. This study observed elevated serum TREM-1 and PGLYRP1 levels in RA and BD groups compared to HCs, with significantly higher levels in RA than BD and HCs. Furthermore, a positive correlation between TREM-1 and PGLYRP1 serum levels and clinical disease activity were observed in RA patients. Earlier studies have also found elevated TREM-1 levels in synovial fluid, plasma and serum of RA patients compared to controls with some of them reporting significant associations to clinical and laboratory parameters of RA disease activity, although the relationship of TREM-1 to radiological damages or the functional capacity has not been discussed^12–14,16^.

Thus far, to our knowledge, only two studies have investigated serum levels of TREM-1 relative to MTX treatment in RA patients. In contrast to Gamez-Nava et al.^48^, who failed to identify differences in serum TREM-1 levels relative to MTX use, our findings demonstrated significantly decreased serum TREM-1 levels in RA patients on MTX as compared to those on other csDMARDs. Similar trends were observed in RA patients stratified by the absence or presence of periodontitis. Among participants without periodontitis, RA patients on MTX tended towards lower levels of TREM-1 compared to non-MTX, although these differences remained below significance. Furthermore, in a recent experimental setup, MTX and oral bactericidal and antibiotic treatments were found comparable in their amelioration of alveolar bone loss, with MTX also efficiently decreasing serum levels of receptor activator of NF-κB ligand (RANKL) in RA-patients^49,50^. As *P*. *gingivalis* was shown to stimulate alveolar bone loss by inducing RANKL expression, the study hypothesized that the beneficial effect of MTX on alveolar bone loss might be mediated via RANKL^51,52^.

Also, to the best of our knowledge, the current report evaluated for the first time the effect of MTX on serum PGLYRP1 levels in RA patients. Our results indicate that neither MTX treatment nor periodontal status were significantly associated with variations in PGLYRP1 serum levels.

TREM-1 and PGLYRP1 levels in various biofluids are elevated in periodontitis patients compared to periodontally-healthy controls^24–29^. A recent study investigating 92 inflammatory biomarkers in serum of RA patients with and without periodontitis, identified 17 markers which were significantly elevated in serum of RA patients with periodontitis compared to periodontally-healthy RA patients^53^. The two inflammatory molecules investigated here, TREM-1 and PGLYRP1, followed the same trend. Both serum TREM-1 and PGLYRP1 levels were increased in RA patients with periodontitis compared to RA patients with healthy periodontium, supporting a potentiating effect of periodontitis towards RA disease activity and inflammation. Similar findings were observed in a previous study investigating the link between periodontal and systemic diseases, revealing higher levels of salivary TREM-1 in chronic kidney disease patients with concomitant periodontitis than periodontally-healthy controls^28^. However, a recent study reported a non-significant association between periodontitis and RA in patients aged < 60 years, while a significant association between tooth loss and RA was found^54^. Although dental caries and periodontitis are primary causes of tooth loss^55^, this study revealed the association between RA and tooth loss independent of dental caries. They concluded that some other factors might influence the association between RA and tooth loss, such as reduced salivary flow rates in RA patients^56^.

We further observed a higher prevalence of periodontitis, higher total bacterial load and significantly increased tooth loss in the RA group (38%) as compared to BD (22%) and HC (7%). These findings are in line with evidence indicating that RA patients are at increased risk of developing periodontitis^33^. Furthermore, a mediation analysis, was carried out to evaluate both direct and indirect causal effects among dependent, independent and mediator variables^35^. In our mediation model, the increased TREM-1 concentration found in RA statistically appeared directly mediated by the presence of concurrent periodontitis. Of note, however, potential influences by other factors could not be completely ruled out in this statistical model.

In patients with BD, both TREM-1 and PGLYRP1 serum levels were found to be significantly lower compared to RA patients, whereas BD patients bared higher TREM-1 levels then HCs. These results are in line with a previous study that also reported elevated TREM-1 levels in BD patients when compared to healthy controls^9^. In addition, the DMFT score was positively correlated with TREM-1 and PGLYRP1 levels in BD. Poor oral health has been reported to be a mediator variable for disease severity in patients with BD^34^.

One potential limitation of the current study might stem from demographic variables, which demonstrated an overrepresentation of female patients in the RA group. RA patients were also older than other participants (mean age 49 years). Nonetheless, whereas RA is acknowledged to be 2–4 times more prevalent in females than in males^57,58^, these demographics are also in-line with previous studies that showed associations between age (especially early post-menopausal period) and the development of RA, thereby owing an explanation to our observations^59,60^.

## Conclusions

Serum TREM-1 and PGLYRP1 levels correlated with RA disease activity, and serum TREM-1 levels were significantly lower in MTX-treated RA patients. In addition, serum TREM-1 and PGLYRP1 levels were associated with poorer oral and periodontal health. Therefore, disease control with csDMARDs treatment, primarily MTX, and protection of oral health with regular dental check-ups might contribute to reduce oral inflammation and beneficially affect the course of RA.

## Supplementary Information

Supplementary Figure 1.

## Data Availability

The datasets generated and analyzed during this study are included in this published article or available from the corresponding author on reasonable request.
