# Supplementary material for: Elevated serum TREM-1 is associated with periodontitis and disease activity in rheumatoid arthritis
Source: Sci Rep. 2021 Feb 3;11:2888. doi: 10.1038/s41598-021-82335-9 (PMC7859204; doi:10.1038/s41598-021-82335-9)
Supplement: Supplementary file 1 — Supplementary Figure 1. [file 41598_2021_82335_MOESM1_ESM.docx]

**ELEVATED SERUM TREM-1 IS ASSOCIATED WITH PERIODONTITIS AND DISEASE ACTIVITY IN RHEUMATOID ARTHRITIS**

**Nevsun Inanc^1^, Gonca Mumcu^2^, Meryem Can^3^, Meral Yay^4^, Angelika Silbereisen^5^, Daniel Manoil^5^, Haner Direskeneli^2^, Nagihan Bostanci^5^**

^1^ Department of Internal Medicine, Division of Rheumatology, School of Medicine, Marmara University, İstanbul, Turkey

^2^ Department of Health Management, Faculty of Health Sciences, Marmara University, İstanbul, Turkey

^3^ Department of Internal Medicine, Division of Rheumatology, School of Medicine, Medipol University, İstanbul, Turkey

^4^ Department of Statistics, Mimar Sinan Fine Arts University, Istanbul, Turkey

^5^ Division of Oral Diseases, Department of Dental Medicine, Karolinska Institutet, Stockholm, Sweden

**SUPPLEMENTARY MATERIAL**

**Supplementary Figure 1:**

**TREM-1 and PGLYRP1 serum levels in control groups.** **A**; serum levels of TREM-1 and PGLYRP1 are compared between HC participants affected or not by periodontitis. **B**; serum levels of TREM-1 and PGLYRP1 are compared between BD patients affected or not by periodontitis. Boxplots show the first and third quartiles (top and bottom edges of the rectangle) divided by the median. Whiskers correspond to the highest and lowest values. A base-10 log scale is applied on the y-axis. HC; healthy control, BD; Behçet disease, ns; non-significant.
